# Triple emergencies: Hyperosmolar hyperglycemic state, venous thromboembolism, and huge free‐floating right heart thrombus successfully managed with anticoagulation

**DOI:** 10.1002/ccr3.4710

**Published:** 2021-12-26

**Authors:** Hayatu Umar, Usman Muawiyya Zagga, Femi Akindotun Akintomide, Abdulaziz Aminu, Abubakar S. Maiyaki, Umar Zulkifilu, Musa Tambuwal Umar, Kabiru Mande Muhammad, Adeshina Abdulateef Yusuf, Adamu Jibril Bamaiyi

**Affiliations:** ^1^ Department of Internal Medicine Usmanu Danfodiyo University Teaching Hospital, Usmanu Danfodiyo University Sokoto Nigeria; ^2^ Department of Physiology, Faculty of Clinical sciences Usmanu Danfodiyo University Sokoto Nigeria

**Keywords:** huge right heart thrombus, hyperosmolar hyperglycemic state, low molecular weight heparin, venous thromboembolism, warfarin

## Abstract

A 57‐year‐old man, with type 2 diabetes mellitus, was admitted with a hyperosmolar hyperglycemic state, who developed in‐hospital venous thromboembolism with huge free‐floating right heart thrombus, and there is no available optimal treatment option for the huge free‐floating right heart thrombus, except anticoagulation with warfarin and low molecular weight heparin with successful outcome.

## Graphical abstract

In a resource‐constrained setting, therapeutic anticoagulation though less favored may be helpful with a favorable outcome in the absence of an optimal therapeutic strategy in patients with HHS who develop VTE and right heart thrombus.

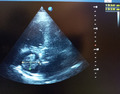

## 1 INTRODUCTION

Diabetes mellitus (DM) is a multisystemic disease characterized by hyperglycemia, which is regarded as a prothrombotic factor and hypercoagulable state.^1^, ^2^ Thus, it is a common risk factor for stroke, coronary artery disease, peripheral arterial disease, and venous and arterial thrombosis. The pathophysiologic mechanisms behind this phenomenal state arise mainly from endothelial dysfunction, platelet hyperactivity, coagulation activation, hypofibrinolysis, hypercoagulability, and venous stasis.^2^, ^3^ Hyperosmolar hyperglycemic state (HHS) is an acute life‐threatening complication of type 2 DM, believed to magnify the abovementioned pathophysiological abnormalities by a positive influence on factors VII and VIII activities, tissue factor pathway inhibitors, platelet activation, aggregation, and release of higher platelet products. In addition, dehydration and hyperglycemia increase blood viscosity, contributing to a hypercoagulable state which enhances venous stasis as part of Virchow's triad, leading to thromboischemic events.^1^, ^2^, ^4^, ^5^ In view of the aforementioned pathophysiological processes, and several reported cases of deep venous thrombosis (DVT) with fatal pulmonary embolism (PE) in acute diabetic emergencies,^6^, ^7^ the national HHS guideline recommends low‐molecular‐weight heparin (LMWH) for all HSS patients for the whole duration of their hospital stay.^8^ However, in spite of this guideline, LMWH for thromboprophylaxis is hardly given to patients presenting with HHS in a resource‐constrained setting, probably due to high cost besides therapeutic inertia exhibited by clinicians.^9^

In spite of the hyperosmolar hyperglycemic state being an exaggerated hypercoagulable state with a high risk of thrombosis, formation of thrombi within the right heart chambers is uncommon except when there is an underlying DVT and/or structural heart disease. Type A thrombi, also called right heart thrombi in transit (RHThIT), are dislodged thrombi from the systemic veins that accidentally stopover within the right atrial chamber (freely mobile) on their way to the lungs to manifest eventually as PE.^10^, ^11^ Type B thrombus is formed in situ within the right heart chambers due to background structural or electrical cardiac abnormalities and is usually nonmobile. Type C partly shares the echocardiographic characteristics of both Type A and B thrombi.^11^ Free‐floating thrombi within the right atrium in coexistence with pulmonary embolism are associated with increased mortality of greater than 40%.^12^ Nevertheless, DVT with or without free‐floating right heart thrombi (RHT) in HHS as reported by Shujaat et al.^6^ may result in fatal pulmonary embolism (PE). Therefore, failure to administer thromboprophylaxis may result in VTE and free‐floating RHT in the setting of HHS, which are life‐threatening medical emergencies that require prompt recognition and timely institution of management to avert fatal outcomes.

## 2 CASE PRESENTATION

A 57‐year‐old man with T2DM for about 10 years, who was not regular on medications and clinic follow‐up visits, presented to our emergency room (ER) with a 2‐day history of polyuria, polydipsia, generalized body weakness, and a 1‐day history of restlessness. However, there was no associated fever, convulsion, differential weakness of limbs, dyspnea, cough, paroxysmal nocturnal dyspnea, leg, or abdominal swelling. He neither smokes nor drinks alcoholic beverages.

The examination at the ER revealed a lethargic middle‐aged man with no obvious respiratory or painful distress, not pale, anicteric, acyanosed, afebrile with a temperature of 37.1℃, and no pedal edema, though he was restless and dehydrated. The respiratory rate was 20 cycles/min, and oxygen saturation (SPO2) was 98% at room air. His pulse rate was 92/min, regular, normal volume, blood pressure was 100/80 mmHg, supine position, heart sounds were first (S1) and second (S2), normal, and there was no murmur. The chest was clinically clear. Abdominal and neurological examinations were unremarkable. Admitting random blood sugar was 36 mmol/L, sodium‐144 mmol/L, potassium‐5.2 mmol/L, urea‐8.6 mmol/L, creatinine‐1.3 mg% and calculated plasma osmolality was 332.6 mosmol/L. Urinalysis revealed glycosuria of +++ and proteinuria of +. The full blood count and differentials, and fasting lipid profile were within normal limits. A working diagnosis of HHS was entertained. He was started on intravenous fluid therapy of 0.9% normal saline, IV and subcutaneous soluble insulin, hourly random blood sugar, and correction/maintenance of electrolytes as required. No any form of thromboprophylaxis was instituted.

**FIGURE 1:**
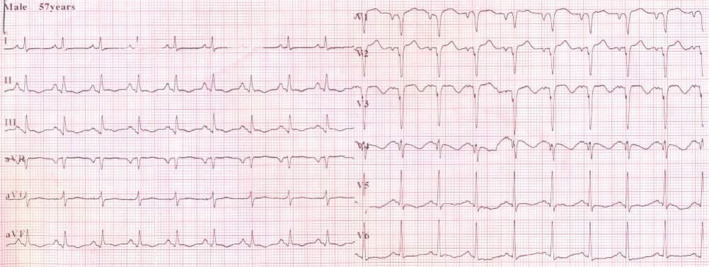
Electrocardiogram showing sinus tachycardia, S1Q3T3 pattern, and slurring of initial negative QRS complex in V2, V3

**FIGURE 2:**
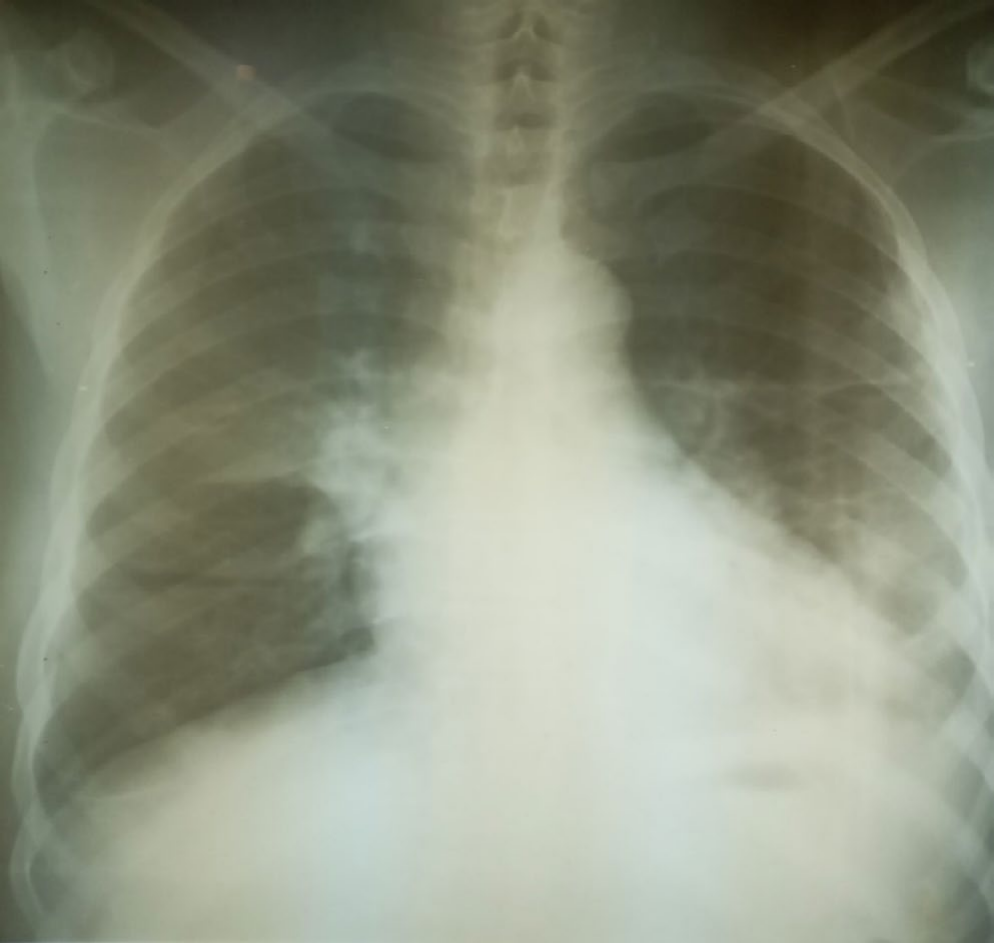
Chest X‐ray showing cardiomegaly, prominent hilar/upper lobe vascular marking, and hazy costophrenic angles

**FIGURE 3:**
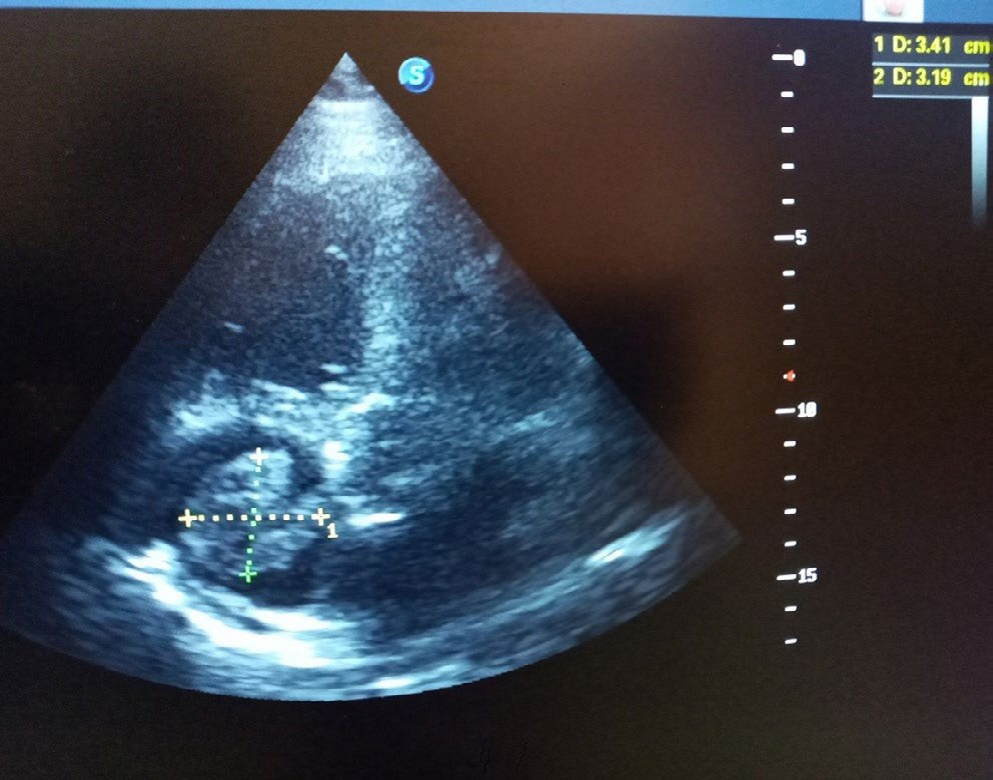
Two‐dimensional (2D) echocardiogram showing huge thrombus in the right atrium, measuring 34.1 × 31.9 mm

On the third day of admission, he developed a sudden onset of severe chest pain, associated with dyspnea and dry cough. However, there was no diaphoresis or feeling of impending doom. The general examination findings revealed that he was in painful and respiratory distress, and respiratory rate was 28 cycle/min, acyanosed with SPO2 of 94% at room air and afebrile (T:36.8℃). There was differential swelling of the right lower limb, shiny, tender, pitting edema, and a circumferential diameter of 5 cm greater than the left lower limb. Except for tachycardia, there was no raised jugular venous pressure or tender hepatomegaly, and the chest was still clinically clear. Calculated Well's pretest probability score for DVT and PE was 2 and 7.5, respectively. The electrocardiogram showed sinus tachycardia with a heart rate of 111 bpm, normal QRS axis, QRS duration‐115 ms, PR duration‐133 ms, corrected QT‐451 ms, S1Q3T3 pattern, and slurring of the initial negative QRS complex in V2, V3, and T‐wave abnormality (Figure 1). Chest X‐ray showed cardiomegaly of bi‐ventricular configuration (CTR:0.54), prominent hilar/upper lobe vascular marking, and hazy costophrenic angles (Figure 2). The echocardiography revealed a dilated left atrium (45.90 mm), right atrium (48.30 mm), and right ventricle with an RV internal diameter at D1 of 30.8 mm. Dilated left ventricle with an internal diameter at diastole of 58.6 mm and left ventricular global hypokinesia with systolic dysfunction (ejection fraction was 25.43%). A huge free‐floating thrombus in the right atrium was seen, and it measured 34.1 × 31.9 mm (Video S1 and Figure 3). There was moderate tricuspid regurgitation, mild pulmonary regurgitation, and pulmonary arterial hypertension (mean pulmonary arterial pressure of 30 mmHg at rest). The echocardiographic conclusion was a suspected PE with a huge free‐floating right heart thrombus. The color Doppler ultrasound scan of the lower extremities showed extensive deep vein thrombosis of the right lower limb: complete occlusive thrombosis of the proximal common femoral and the entire course of the superficial femoral and the popliteal veins. Cardiac markers revealed troponin I and CK‐MB to be 0.463 ng/ml and 10.28 U/L, respectively. The requested computerized tomography pulmonary angiogram and D‐dimer assay were not done due to nonavailability of facilities.

A provisional diagnosis of venous thromboembolism with huge RHT was considered. He was immediately commenced on subcutaneous LMWH: Enoxaparin 1 mg/kg 12 hourly for 72 h, thereafter 1.5 mg/kg daily for 4 days in conjunction with warfarin at 5 mg daily with an international normalized ratio (INR) target of 2–3. 100% oxygen was administered via a face mask. In view of the extensive DVT and huge RHT, he was counseled for interventional/surgical procedures in a center with the expertise, which he declined due to financial constraints. Consequently, we continued the only available therapeutic option, which is anticoagulation with heparin and warfarin despite the low success rate, especially in the management of free‐floating RHT, although useful in the management of coexisting DVT and suspected PE.

A follow‐up echocardiogram performed a week after commencement of anticoagulation demonstrated no residual free‐floating thrombus in the right atrium (Video S2). Pulmonary arterial pressure was normal, and EF was 61%. The patient achieved great recovery with fasting blood sugar of 5.2 mmol/L, oxygen saturation was 98%–99% at room air, and there were no observed in‐hospital hemorrhagic complications. Captopril was added to his antidiabetic and warfarin prior to discharge. He was encouraged to adhere to his medications, regular clinic follow‐up, and ensure regular INR to monitor efficacy and compliance with anticoagulant. Currently, he is in good health.

## 3 DISCUSSION

In this case report, we highlight the inertia in prescribing LMWH for DVT prophylaxis, which resulted in the uncommon coexistence of the triple life‐threatening medical emergencies: HHS with VTE and a huge free‐floating RHT, and the incidental finding of left ventricular dilatation, global hypokinesia, and systolic dysfunction, where the free‐floating RHT was managed with therapeutic anticoagulation, thus with favorable outcome in a resource‐constrained setting. Free‐floating RHT is an amazing phenomenon when observed in real‐time echocardiography, this is associated with grave danger in vivo as reported in 4%–18% of cases of venous thromboembolism,^13^, ^14^ and failure to recognize it promptly so as to institute emergent management has been reported to lead to significant mortality in the range of 27%–100%.^13^, ^15^, ^16^ The right atrium is the site of predilection where mobile thrombus is commonly seen in 79%, as in our index case, than the right ventricle, 16%.^15^ Although HHS is a prothrombotic factor and hypercoagulable state as stated by Lemkes BA and Pomero F. et al.^1^, ^2^ with multiple thrombotic tendencies as reported by Ganaw AE et al.^17^ Irrespective of the aforementioned pathophysiologic events in HHS, formation of RHT with the potential risk of embolization is rare in patients presenting with HHS, except when there is an underlying DVT and/or structural heart disease as noted in the index case. The echocardiographic finding of left ventricular dilatation with global hypokinesia and systolic dysfunction prompted consideration of background diabetic, ischemic, or idiopathic dilated cardiomyopathy as possible etiologies. Notably, there were no symptoms to suggest heart failure prior to admission. However, VTE being an acute event may lead to left ventricular systolic dysfunction but is unlikely to cause left ventricular dilatation and global hypokinesia.

The optimal therapy for free‐floating RHT is still controversial. Nevertheless, favored therapeutic options include thrombolysis, surgical thromboembolectomy, percutaneous transcatheter clot retrieval, or directed thrombolysis technique, whereas anticoagulation is not considered as an optimal therapeutic option^18^; unfortunately, this is the only available choice in our setting, more so because our patient is financially constrained to access optimal care. Nonetheless, heparin and warfarin can be used, albeit at a low recommendation, hence the choice of this option in our index patient.

## 4 CONCLUSION

In conclusion, this case, therefore, re‐emphasizes the need to give thromboprophylaxis to patients with HHS so as to avert the uncommon coexistence of the triple emergencies: venous thromboembolism with associated huge free‐floating RHT in HHS, which can invariably result in grave outcomes if not detected early with a view to treatment even with the less proven therapeutic option of anticoagulation in poor resource settings.

## CONFLICT OF INTEREST

None declared.

## AUTHOR CONTRIBUTIONS

All authors gave final approval and agreed to be accountable for all aspects of the case report. **HU** contributed to patient care, conception of work, drafting, interpretation of findings, performed echocardiography, drafting, and obtained informed consent. **UMZ** contributed to patient care, conception of work, drafting, and interpretation of findings, performed echocardiography, and obtained informed consent. **FAA** contributed to conception of work, drafting, interpretation of findings, critically revising work, and editing of photographs and legends. **AA:** contributed to patient care, extracting history from case note, obtaining the figures and videos, performed echocardiography, and obtained informed consent. **ASM** contributed to conception of work, interpretation of findings, critically revising work, and editing of photographs and legends. **ZU** contributed to patient care, extracting clinical history from case note, and obtaining the figures and videos and obtained informed consent. **MTU** contributed to patient care, drafting, interpretation of findings, and revising critically and obtained informed consent. **KMM:** contributed to conception of work, extracting history from case note, obtaining the figures and videos, and critically revising. **AAY** contributed to conception of work, extracting history from case note, obtaining the figures and videos, and critically revising and obtained informed consent. **AJB** contributed to conception of work, drafting, critically revising, and editing of photographs and figure legends.

## CONSENT FOR PUBLICATION

Written informed consent was obtained from the patient for publication of this case report along with images and videos.

## Supporting information

Video S1

Video S2

## Data Availability

The data that support the findings of this study are available from the corresponding author upon reasonable request.
